# Bone Density Micro-CT Assessment during Embedding of the Innovative Multi-Spiked Connecting Scaffold in Periarticular Bone to Elaborate a Validated Numerical Model for Designing Biomimetic Fixation of Resurfacing Endoprostheses

**DOI:** 10.3390/ma14061384

**Published:** 2021-03-12

**Authors:** Ryszard Uklejewski, Mariusz Winiecki, Adam Patalas, Piotr Rogala

**Affiliations:** 1 Chair of Construction Materials and Biomaterials, Institute of Materials Engineering, Kazimierz Wielki University, 85-064 Bydgoszcz, Poland; winiecki@ukw.edu.pl; 2 Laboratory of Bone Implants Research and Design, Department of Technology Design, Institute of Mechanical Technology, Poznan University of Technology, 60-965 Poznan, Poland; adam.patalas@put.poznan.pl (A.P.); gabinet.rogala@gmail.com (P.R.); 3 Institute of Health Sciences, Hipolit Cegielski State College of Higher Education, 62-200 Gniezno, Poland

**Keywords:** multi-spiked connecting scaffold (MSC-Scaffold), biomimetic fixation for resurfacing endoprostheses, micro-CT assessment, periarticular bone density, validated numerical model

## Abstract

Our team has been working for some time on designing a new kind of biomimetic fixation of resurfacing endoprostheses, in which the innovative multi-spiked connecting scaffold (MSC-Scaffold) that mimics the natural interface between articular cartilage and periarticular trabecular bone in human joints is the crucial element. This work aimed to develop a numerical model enabling the design of the considered joint replacement implant that would reflect the mechanics of interacting biomaterials. Thus, quantitative micro-CT analysis of density distribution in bone material during the embedding of MSC-Scaffold in periarticular bone was applied. The performed numerical studies and corresponding mechanical tests revealed, under the embedded MSC-Scaffold, the bone material densification affecting its mechanical properties. On the basis of these findings, the built numerical model was modified by applying a simulated insert of densified bone material. This modification led to a strong correlation between the re-simulation and experimental results (FVU = 0.02). The biomimetism of the MSC-Scaffold prototype that provided physiological load transfer from implant to bone was confirmed based on the Huber–von Mises–Hencky (HMH) stress maps obtained with the validated finite element (FE) model of the problem. The micro-CT bone density assessment performed during the embedding of the MSC-Scaffold prototype in periarticular bone provides insight into the mechanical behaviour of the investigated implant-bone system and validates the numerical model that can be used for the design of material and geometric features of a new kind of resurfacing endoprostheses fixation.

## 1. Introduction

Among orthopaedic implants, joint replacements have the greatest market share [1]. The hip replacement market has been segmented into total hip implants, partial hip implants, hip resurfacing, and revision hip implant; and among these, total hip implants are poised to provide approximately 58.4% of the global hip replacement market by the end of 2024. In terms of revenue, this segment is anticipated to be valued at USD 5329.4 million by the end of 2024 [2].

In a traditional total hip arthroplasty, the damaged bone and cartilage are removed and replaced with artificial prosthetic components, such as a hip endoprosthesis with a stem and acetabular (socket) cup system. The damaged femoral head is removed and replaced with a metal stem (cemented/non-cemented) that is placed into the femoral canal. In hip resurfacing, the femoral head is not removed but rather trimmed and capped with a smooth metal covering cap. The damaged bone and cartilage within the socket are removed and replaced with a metal shell, just as in a traditional total hip replacement [3].

A hip replacement can be made of numerous materials, such as Ti-alloy, Co-Cr alloy, polyethylene, and poly(methyl methacrylate), all of which exhibit high mechanical strength and wear resistance. Over the past century, different combinations of materials have been developed as hybrid fixation implants. These implants provide sufficient mechanical strength to support the destroyed bones or anatomical structures as well as good wear resistance to realize long-term fixation stability [4,5].

One of the advantages of hip resurfacing over traditional total hip arthroplasty endoprostheses is that the former is easier to revise. If an implant fails, a revision arthroplasty operation is necessary, and it is more complicated than the initial operation. In hip resurfacing arthroplasty, less femur bone is removed as compared with a traditional hip replacement. Thus, many surgeons believe it is easier to exchange implants that fail after hip resurfacing [6].

In the vast majority of cases, cement fixation is the worldwide recognised implant-bone fixation method for femoral components of resurfacing arthroplasty (RA) endoprostheses. To avoid problems with cement (collapse, excessive cement penetration, fatigue failure, potential for thermal necrosis, etc.) [7,8] and accomplish long-term biological fixation, cementless femoral head components appear to be an attractive option.

Our research group designed, developed, and prototyped, through bioengineering research, the essential innovation for a fixation method of RA endoprostheses components in periarticular trabecular bone by means of an innovative multi-spiked connecting scaffold (MSC-Scaffold) [9,10,11,12]. The MSC-Scaffold concept was proposed by a member (orthopaedic surgeon) of the research group [13,14]. The MSC-Scaffold substitutes the short stem of the femoral component of the applied total hip resurfacing arthroplasty (THRA) endoprosthesis with multiple spikes with defined geometry. Our advanced prototype biomimetic implant fixation with bone manufactured by applying additive technology, opens a new generation of the first biomimetic RA endoprostheses, which can be applied for most diarthrodial joint arthroplasties (hip, knee, shoulder, elbow, etc.) used in orthopaedic surgical treatment. This new kind of implant-bone fixation for resurfacing endoprostheses is characterized by biomimetism of the MSC-Scaffold respecting the microstructure of the periarticular subchondral and cancellous bone tissue. The MSC-Scaffold mimics the natural interface (subchondral bone interdigitations) between articular cartilage and periarticular trabecular bone in human joints [15]. Therefore, it provides the close-to-natural load transfer, such as in the biomechanical environment of the natural hip joint, which goes through the bone trabeculae in the head and the neck of the femur, and then along the femoral shaft.

Implantation of calcium phosphate (CaP) surface-modified scaffold prototypes in an animal model has revealed the scaffolding effect [16]. The majority of the interspike pore space of the MSC-Scaffold prototypes was filled by newly formed and properly remodeled bone tissue, providing primary biological fixation of the MSC-Scaffold prototypes in periarticular cancellous bone. A study by [17] was performed on a representative group of animals (swine) and confirmed that the CaP surface-modified MSC-Scaffold allows for entirely cementless fixation of the components of resurfacing arthroplasty (RA) endoprostheses in periarticular cancellous bone with very good clinical stability. Uklejewski et al. determined the suitable range of conditions for CaP potentiostatic electrochemical deposition on the surfaces of spikes of the MSC-Scaffold to achieve a biomineral coating with a native Ca/P ratio [18].

Computational studies on the influence of various geometrical features of an initially embedded MSC-Scaffold on mechanical stresses in peri-implant bone have revealed the features that determine the appropriate MSC-Scaffold design [12]. Before planned experimental surgical treatment with this new type of hip endoprosthesis in humans, it is crucial to develop a validated numerical model for the bioengineering design of this new kind of biomimetic fixation in the bone of resurfacing endoprostheses components.

Combinations of experiments with computational models, especially finite element (FE) models, have commonly been used to study bone and implant mechanics. For example, Enns-Bray et al. [19] used experimental data for the validation of anisotropic finite element models of the proximal femur. Affes et al. [20] featured a pull-out experimental test of screw-bone tibial interface which served as a useful validation for the developed numerical models of the studied problem. Huang et al. [21] used finite element analysis and computer tomography imaging to investigate bone stresses surrounding a dental implant; Du et al. [22] placed dental implants in a human cadaver mandible, and then detailed the geometry of trabecular structures and implants with micro-CT imaging, and subsequently, the stress and strain distributions in the bone under implant loading were computed and compared with prior experimental discoveries. The advantage of micro-CT imaging is that it does not damage a bone sample, and therefore changes in bone structure can be accurately examined [23,24].

In this study, bone density micro-CT assessment during the embedding of the innovative multi-spiked connecting scaffold prototype in periarticular bone is performed to develop a validated numerical model for designing a new kind of biomimetic fixation of resurfacing endoprostheses.

## 2. Materials and Methods

### 2.1. General

**Figure 1:**
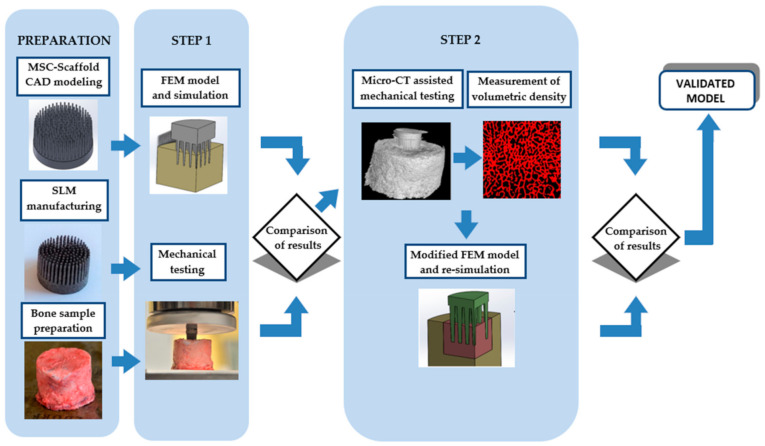
A schematic description of the experimental validation of the finite element (FE) model of the multi-spiked connecting scaffold (MSC-Scaffold) prototype embedded in periarticular bone.

The FE model of the MSC-Scaffold prototypes for RA embedded in periarticular bone was validated in two experimental steps preceded by the preparation tasks, including computer-assisted design (CAD) modelling, selective laser melting (SLM) manufacturing (Section 2.2), and bone sample preparation (Section 2.3). In the first step, the mechanical load transfer from the implant to the periarticular bone was investigated, at different levels of the MSC-Scaffold, for embedding in periarticular bone by applying a built FE model of a Ti-Alloy MSC-Scaffold prototype embedded in an elastic, transversely isotropic bone material (Section 2.4). Then, mechanical tests of embedding the MSC-Scaffold in samples of swine periarticular bone were performed (Section 2.5). In the second step, micro-CT scanning of periarticular bone samples and micro-CT assisted mechanical tests were performed for quantitative analysis of the density distribution of the bone material before and during the mechanical embedding process (Section 2.6). The obtained data from the micro-CT imaging were used to modify the FE model and to perform subsequent simulation studies (FEM re-simulation, Section 2.7). For validation of the built model, the correlation between experimental and numerical results was analysed (Section 2.8). A schematic description of the experimental validation is shown in Figure 1.

### 2.2. Computer-Assisted Design (CAD) Modelling and Selective Laser Melting (SLM) Manufacturing of Multi-Spiked Connecting Scaffold (MSC-Scaffold) Prototypes

**Figure 2:**
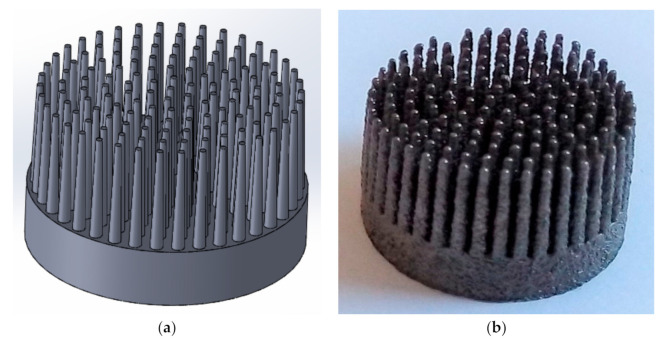
(**a**) Computer-assisted design (CAD) model of MSC-Scaffold prototypes for RA endoprostheses; (**b**) The MSC-Scaffold prototype selective laser melting (SLM) manufactured of Ti-6Al-4V alloy on the base of the CAD models.

During CAD modelling of the MSC-Scaffold prototype, directives from the previous works [11,12] were taken into account. The multilateral spikes of the MSC-Scaffold, which were shaped like a truncated cone with a height of 5 mm, were arranged in concentric parallel rings around the central spike (with axes parallel to each other). The central spike was coincident with the femoral head axis of symmetry. The diameter of the base of the spikes in the MSC-Scaffold CAD model was 0.5 mm. The distance between the bases of neighbouring spikes was 0.35 mm, both circumferentially and radially, which corresponded to the thickness of cancellous bone trabeculae. The CAD model of the MSC-Scaffold prototype for RA endoprostheses is shown in Figure 2a.

The manufacturing was subcontracted to the Centre of New Materials and Technologies at the West Pomeranian University of Technology in Szczecin, Poland. The following process parameters were applied during the SLM manufacturing: laser power, 100 W; layer thickness, 30 µm; laser spot size, 0.2 mm; scan speed, 0.4 m/s; laser energy density, 70 J/mm^3^. The SLM method was chosen because it is characterised by high precision in the manufacturing of porous or lattice structures [25,26].

To remove the adhered powder aggregates from the spike surfaces after manufacturing, a manual blasting treatment was performed with an experimentally customised abrasive mixture. It was composed of equal proportions of F220 white aloxite (~53–75 μm), F320 white aloxite (~29.2 μm ± 1.5%), and blasting micro glass beads (~30 μm ± 10%). The surfaces were cleaned in an ultrasonic bath (Sonic 3, Polsonic, Warszawa, Poland) using demineralised water with surfactants. The manufactured MSC-Scaffold prototype is shown in Figure 2b.

### 2.3. Periarticular Bone Samples Preparation

Hip joints of Polish Large White swine (aged between 8 and 10 months) were purchased from a local slaughterhouse (Zakład Rzeźniczo-Wędliniarski Edmund Koczorowski, Połajewo, Poland). A swine bone was used because it is a good animal model for human bone. Indeed, the mechanical properties of swine articular bone are consistent with those of human articular bone [27]. The femoral head of each joint was cut out and mechanically cleaned of all soft tissues, wrapped in tissue soaked in Ringer’s solution, sealed in a plastic bag, and stored at 4 °C. The mechanical tests were performed on the fresh bone (i.e., up to 5 h after resection). The cylindrical samples (ø26 mm × 20 mm) were cut using a saw and hole cutter. The rotation diamond wheel saw (IsoMet™ 4000 Linear Precision Saw, Esslingen am Neckar, Buehler, Germany) was used to remove cartilage slices to expose the subchondral bone with unbroken trabeculae.

### 2.4. Finite Element (FE) Model and Simulations

For the FE model simulation, the CAD models of the MSC-Scaffold prototypes were adopted, as described in Section 2.2 and previous simulation studies [12]. The simulated bone element was designed in the shape of a cylindrical section. This geometry was a matrix of the CAD-modelled MSC-Scaffold prototype that reflected the embedded MSC-Scaffold prototype in the periarticular bone.

**Figure 3:**
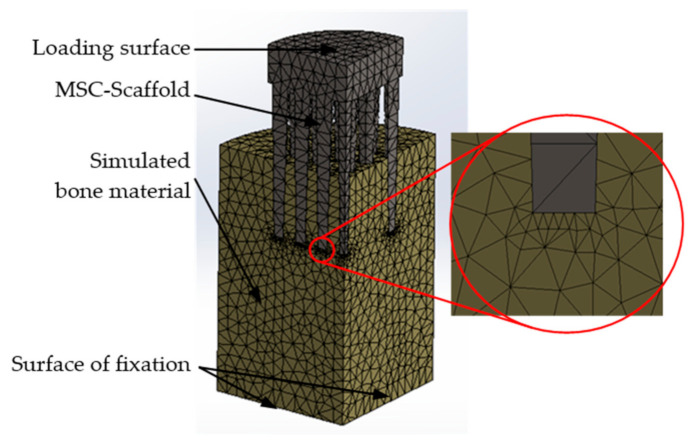
Simulation model of the MSC-Scaffold prototype embedded in the periarticular bone with the generated FE mesh. The surfaces of FE fixation and the loading surface are marked with arrows.

Due to the symmetry of the model, computational studies were performed for a quarter of the built FE model. The base of the bone material cylinder was assumed to be a fixed support (i.e., the bone cylindrical base was constrained in all directions). On the symmetry planes of the model, translations of the lateral surfaces in the normal directions were excluded. Contacts between the MSC-Scaffold and simulated bone material were set, and an augmented Lagrangian method was applied to the contact surfaces (contact penetration was present but controlled). This method is applicable for any type of contact behaviour and is commonly used for symmetric and asymmetric contacts, which are recommended for general frictionless or frictional contacts [28,29]. Figure 3 presents the simulation model of the MSC-Scaffold prototype embedded in the periarticular bone. A mesh convergence study was performed to ensure adequate numerical convergence of the results.

The simulated bone material was assumed to be a single-phase transversally isotropic elastic material. The values of the mechanical properties of the MSC-Scaffold [26,30] and bone material [31,32,33] are presented in Table 1 and Table 2, respectively.

Computational studies were performed to analyse the force required to embed the MSC-Scaffold in the periarticular bone at five different embedding levels. The force needed to embed the MSC-Scaffold in the periarticular bone was assumed to cause the limit stress value determined as the mean of the internodal stresses of the maximum values on the surface contacting with the apexes of the spikes. Additionally, the stress distribution was determined according to the Huber–von Mises–Hencky (HMH) theory.

### 2.5. Mechanical Tests

**Figure 4:**
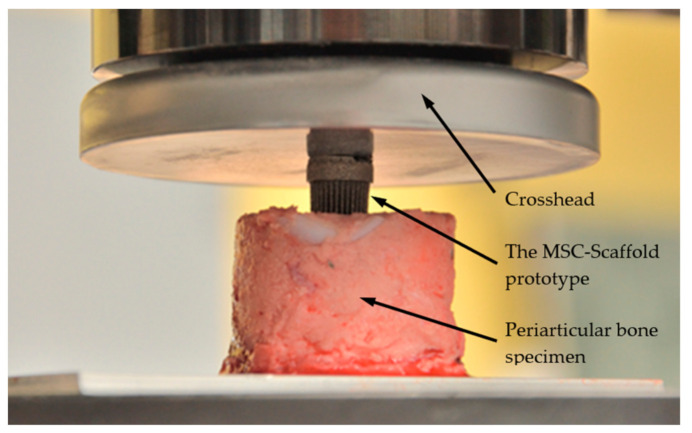
The experimental stand with a bone-implant specimen prepared for the MSC-Scaffold prototype quasi-static embedding tests.

Mechanical tests of the MSC-Scaffold prototype quasi-static embedding in the swine periarticular bone samples were performed with a universal testing machine (Instron 300DX, Instron, Norwood, MA, USA). Emery paper (120 grit) was fixed to the grips, which stabilised the bone sample and protected against slipping. The MSC-Scaffold prototype was placed on the bone sample and preloaded to reach the point of contact (bone-implant specimen). The MSC-Scaffold prototype quasi-static embedding tests were performed at the crosshead speed of 0.1 mm/s until the spikes of the prototypes reached the depth of 3.0 mm in the bone. Quasi-static loading of the prototype scaffold at a rate of 0.1 mm/s corresponds to bone elastic strain rates induced during peaceful gradual loading of an operated limb in the course of postoperative rehabilitation [34,35]. During the tests, the embedding force and the crosshead displacement were measured. Figure 4 shows the experimental stand with a bone-implant specimen prepared for testing.

### 2.6. Micro-CT-Assisted Mechanical Test

**Figure 5:**
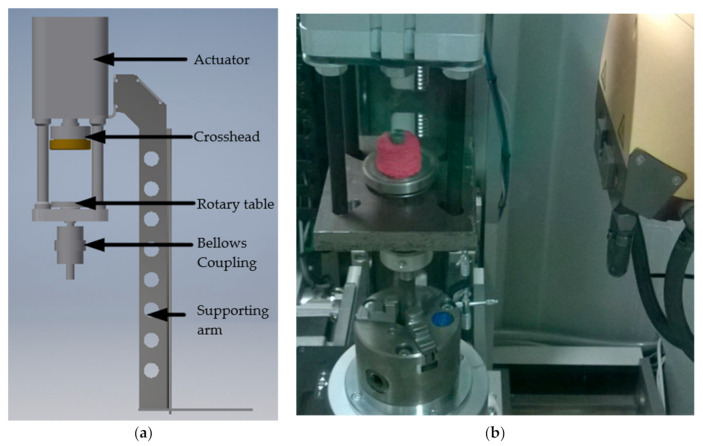
(**a**) The CAD model of the executive system that enabled mechanical tests; (**b**) A representative bone-implant specimen in the chamber of the micro-CT scanner.

Micro-CT-assisted mechanical tests were conducted using a micro-CT scanner (GE phoenix v|tome|x S, GE Measurement and Control, Billerica, MA, USA) equipped with a specialised device that enabled mechanical tests designed and manufactured by our research team. This technique was performed to evaluate the density of bone material before testing. The CAD model of the executive system that mediated mechanical testing is presented in Figure 5a. A picture of the bone-implant specimen in the chamber of the micro-CT scanner is presented in Figure 5b.

**Figure 6:**
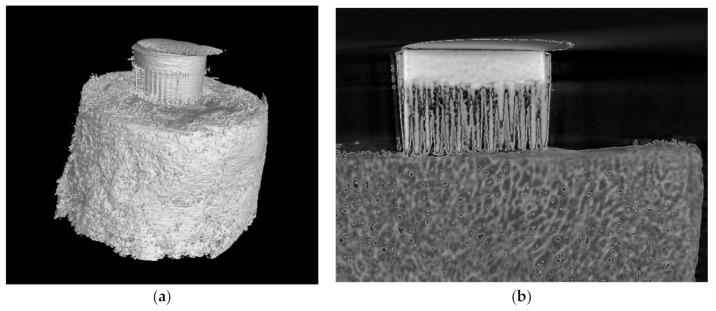
(**a**) The three-dimensional (3D) view of the bone-implant specimen; (**b**) The representative two-dimensional (2D) slice of the micro-CT reconstructed specimen before tests.

The bone-implant specimens were mounted on a rotary stage and scanned in their entirety. The scanning parameters were as follows: source voltage, 130 keV; source current, 125 mA; resolution, 17.5 μm; filter, 1.5 mm brass; exposure time, 300 ms; rotation, 180°, every 0.5°; scanning time, 20 min. The three-dimensional (3D) visualization and two-dimensional (2D) image analysis of the micro-CT reconstructed bone samples and bone-implant specimens were performed using dedicated micro-CT software. In the bone-implant 3D reconstructions, the area of interest was extracted for qualitative and quantitative analyses. The MSC-Scaffold prototype and trabecular bone were identified and distinguished based on radiological density. The soft tissue, including bone marrow, was hidden. A representative view of the reconstructed bone-implant specimen from the micro-CT examination is shown in Figure 6.

For each level of embedding, trabecular bone areas of interest were chosen from the top of the apex spikes of the MSC-Scaffold. Vascular canals and osteocyte lacunae were extracted from the volume of interest using VG Studio Max software [36] and were not subject to further analysis. Compartments that represented porosities were created by selecting all voxels with a radiological density value lower (inverse segmentation) than the threshold values previously determined using the threshold methods. This action produced large objects (canals), small objects (lacunae), and noise. Using a 3D region-growing operation, 1-voxel-sized noise objects were removed using erosion-dilation procedures. To prevent the addition or removal of voxels on edges and boundaries of all segmentations, voxel-based operations were limited to the surfaces of the original thresholded images. Additionally, to prevent edge effects, the first and last images of the volume of interest stack were discarded from the 3D analysis.

Cancellous bone tissue comprises a network of trabeculae and soft tissues that fill the intertrabecular space. The trabecular network and soft tissue are naturally combined in highly variable proportions in different skeletal regions. Thus, one cannot take a fixed reference to approach the volumetric trabecular bone density and have a wide range of values [37,38]. Therefore, to determine volumetric trabecular bone density ($\rho_{T}$) the density of the trabecular network ($\rho_{T}$), and marrow and soft tissue density ($\rho_{w}$) were measured. The volumetric periarticular bone density (*ρ_b_*) was calculated using Equation (1) as follows:

(1) $$\rho_{b}=\;\left(1-\varphi \right)\;\cdot \;\rho_{T}+\varphi \cdot \rho_{w}$$

where:

*ρ_T_* is the volumetric trabecular bone density (comparable to cortical bone and equal to 1.90 g/cm^3^);

*ρ_w_* is the marrow and soft tissues density (similar to water, i.e., 1.00 g/cm^3^);

*φ* is the marrow and soft tissue fraction.

1 − *φ* is the trabecular bone fraction.

Longitudinal Young’s modulus, *E*_2_, of periarticular bone in the regions of interest was calculated on the base of knowing bone density $\rho_{b},$ according to the Equation (2) as follows:

(2) $$E_{2}=315\rho_{b}{}^{3}\;(\mathrm{MPa})$$

### 2.7. Modification of the FE Model and Re-Simulation

**Figure 7:**
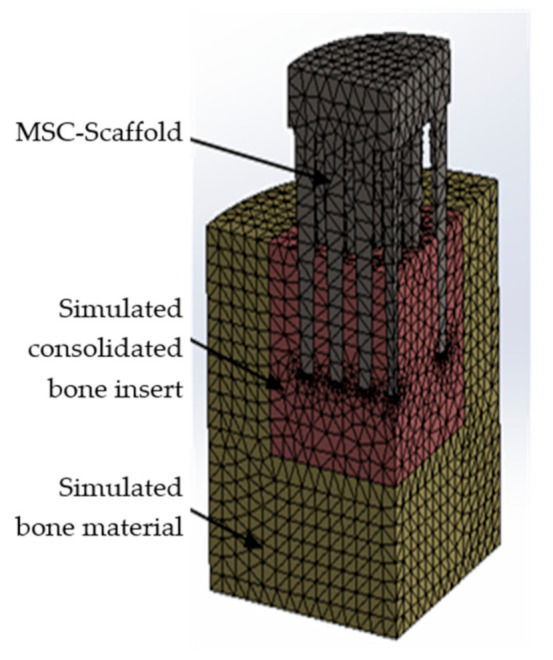
The modified FE model of the MSC-Scaffold prototype embedded in the periarticular bone with the simulated insert of densified bone material (according to micro-CT findings).

The simulated insert of densified bone material was introduced in the FE model. The mechanical properties of this insert were evaluated based on micro-CT imaging and the values of cancellous bone mechanical properties were calculated, as described in Section 2.4. The modified CAD model of the MSC-Scaffold prototype embedded in the simulated periarticular bone and the simulated insert of densified bone material (with a real effective density) is shown in Figure 7.

### 2.8. Analysis of Correlation

All values of experimental results are expressed as means ± standard deviation (SD). Any differences with *P* < 0.05 were considered significant.

The linear regression model was used to analyse and compare experimental and simulation results. The fraction of variance unexplained (FVU) statistical test was performed to determine what part of the explanatory variable variation observed in the sample did not match the model [39,40]. The FVU takes values from the interval {0,1}. The better correlation of the model, the closer FVU is to zero. It is expressed by Equation (3) [41,42] as follows:

(3) $$FVU=\sum_{i=1}^{n}\frac{{\left(y_{i}-{\hat{y}}_{i}\right)}^{2}}{\sum_{i=1}^{n}{\left(y_{i}-{\bar{y}}_{i}\right)}^{2}},$$

where $y_{i}$ represents the empirical value of dependent variable Y at *i*-th value;$\;{\hat{y}}_{i}$ represents the theoretical value of the explanatory dependent variable Y at *i*-th value;$\;\mathrm{and}\;\bar{y_{i}}$ represents the arithmetic means of the empirical values of the variable.

## 3. Results

### 3.1. Mechanical Testing and Initial FE Model Simulations

**Figure 8:**
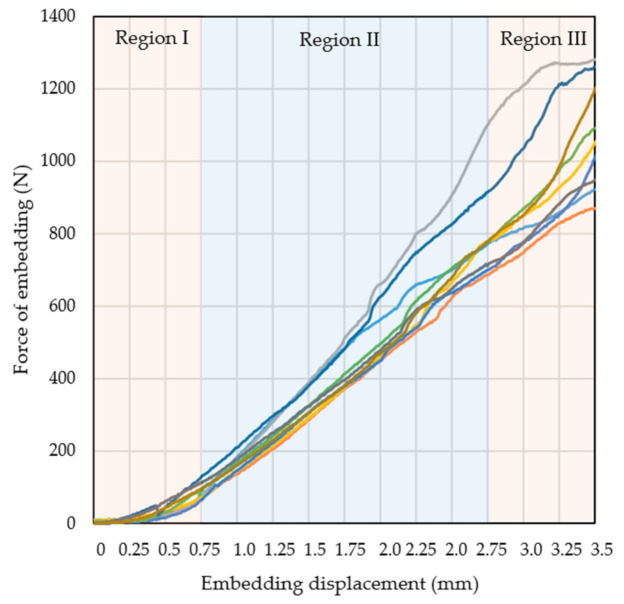
The embedding force-distance curves for 10 representative tests of the MSC-Scaffold prototype embedding in the periarticular bone.

Figure 8 shows the embedding force-distance curves from 10 representative tests of the MSC-Scaffold prototypes embedding in the periarticular bone. The force of embedding is the force that causes the MSC-Scaffold prototypes embedding in the periarticular bone.

The embedding force–distance curves could be categorised into three regions. Region I, the initial phase of embedding the MSC-Scaffold into the periarticular bone, was characterised by a slight increase in the embedding force. Spikes of the MSC-Scaffold penetrated the intertrabecular space of the periarticular bone, and the embedding force increased as the spikes gradually made contact with the trabeculae. Region II showed the linear increase in the embedding force from the partially embedded MSC-Scaffold in the periarticular bone and the spikes in contact with the trabeculae. During this phase, the load was transferred from the spikes of the MSC-Scaffold prototype to the trabeculae of cancellous bone, a phenomenon that caused elastic deformation of this bone. Region III was characterised by embedding force changes due to the destruction of particular trabeculae and the densification of the trabecular bone.

**Figure 9:**
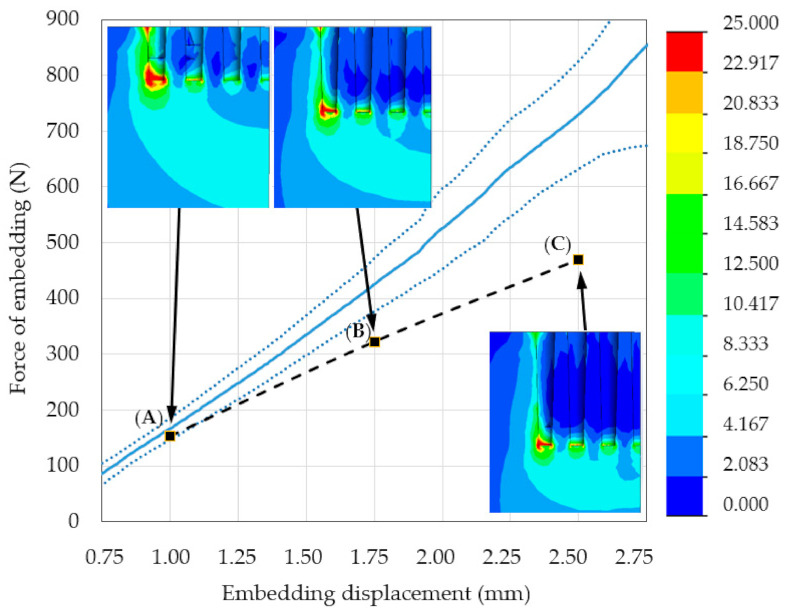
Region II of the force–distance curves obtained from experimental measurements (the mean line is presented as the solid blue line, dotted blue lines are ± standard deviations) and from numerical simulations. The dashed black line represents results of numerical simulation of the problem. The insets show representative maps of the Huber–von Mises–Hencky (HMH) stress distributions calculated for the considered system containing the MSC-Scaffold prototype and periarticular bone material with the embedding load applied to the top surface of the MSC-Scaffold prototype. (**A**) 1 mm embedding displacement and 153 N force of embedding; (**B**) 1.75 mm embedding displacement and 322 N force of embedding; (**C**) 2.5 mm embedding displacement and 469 N force of embedding.

The results of embedding tests obtained in region II were averaged and presented as a mean line (Figure 9). The linear regression model was applied to the mean line and the following parameters were obtained: a = 376.49, b = 209.46, and R^2^ = 0.9932. The high value of the determination coefficient, R^2^, indicates a strong linear relationship.

Figure 9 shows region II of the force–distance curves obtained from experimental measurements and numerical simulations. Representative maps of the HMH stress distributions calculated for the considered MSC-Scaffold and bone system under the force of embedding are inset at several points.

For each analysed scenario, the areas of stress concentration in the periarticular bone were localised around the spike apexes (i.e., the periapical region, Figure 9A–C). Each HMH stress map demonstrated that the reach of the submaximal load limit occurred by embedding displacement by the MSC-Scaffold. These data indicate that the stress builds up around the top of spikes of the MSC-Scaffold. This phenomenon results in less accumulation of elastic energy in the periarticular bone in interspike spaces and below the spikes. There was a discrepancy between the experimental bone-implant embedding tests and the numerical FEM simulations. The value of the FVU statistic test was 0.33, which indicates an insufficient correlation between experimental and numerical data.

### 3.2. Modified FE Model Validation

**Figure 10:**
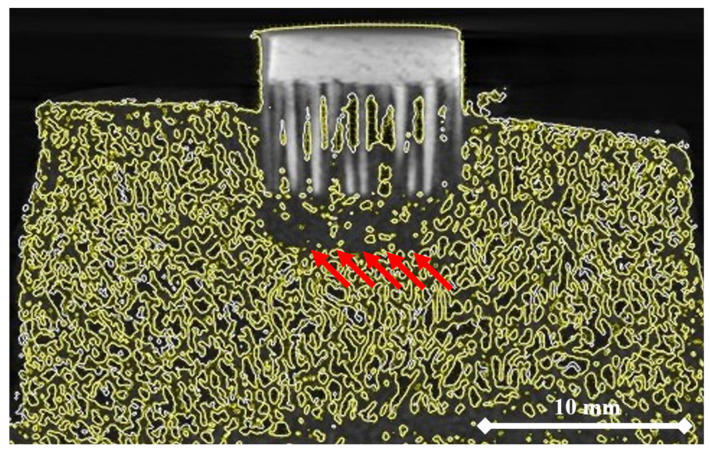
Micro-CT reconstructed specimen of bone with the embedded MSC-Scaffold prototype. The areas of bone material densification are marked with red arrows.

Figure 10 shows the micro-CT reconstructed specimen of the MSC-Scaffold prototype embedded in the periarticular bone. The area of bone densification below the spike apexes is marked with red arrows.

**Figure 11:**
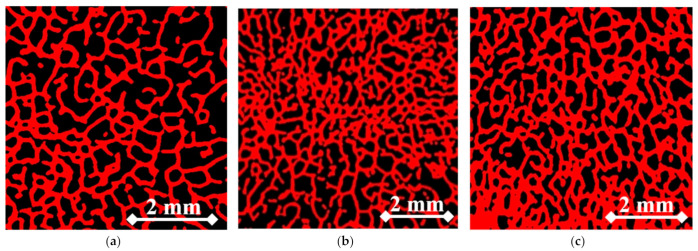
Representative micro-CT slices of bone material below the MSC-Scaffold at different levels of its embedding. (**a**) 1.5 mm; (**b**) 2.0 mm; (**c**) 2.5 mm.

Figure 11 shows representative areas of interest in the bone material, along with a fraction of marrow and soft tissue. They were used to determine trabecular bone density of the periarticular bone and subsequently Young’s modulus. As the embedding displacement of the implant increased, the degree of densification of the bone material also increased. Consequently, there was a reduction in the fraction of marrow with soft tissue and an increase in Young’s modulus (Table 3).

**Figure 12:**
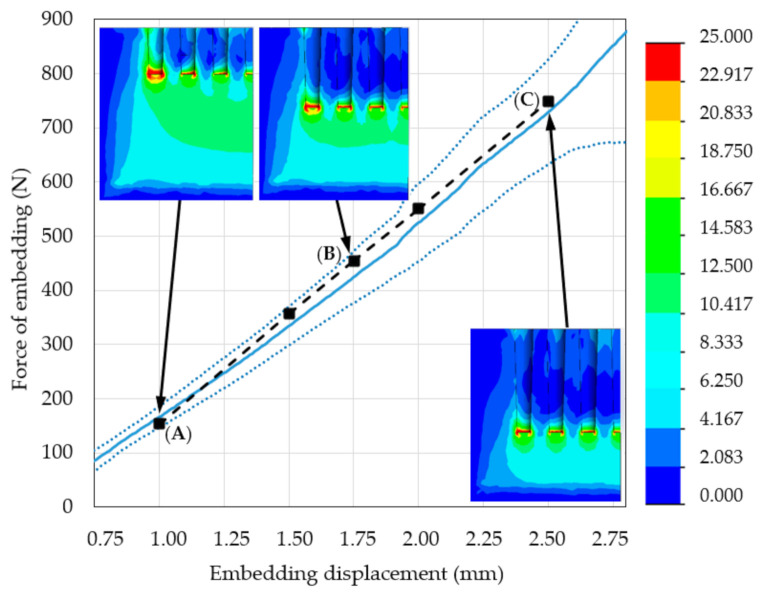
Region II of the force–distance curves obtained from experimental measurements (the mean line is presented as the solid blue line, dotted blue lines are ± standard deviations) and the results of re-simulations with FE model modified by the simulated insert of densified bone material (dashed black line) at (**A**) 1.00 mm embedding displacement and 153 N loading force; (**B**) 1.75 mm embedding displacement and 453 N loading force; (**C**) 2.50 mm embedding displacement and 748 N loading force.

Figure 12 shows region II of the force–distance curves obtained using experimental measurements and re-simulations with an insert of simulated densified bone material. The mechanical properties of this insert, at each level of embedding, corresponded with the values in Table 3. The results for both methods were similar and highly repeatable, a phenomenon that suggests the simulation model is suitable for studying such cases.

According to the stress distribution on the HMH stress maps from the re-simulation study, the stress gradient between the periapical surface of the spikes and the surrounding space decreased. This alteration resulted in a greater accumulation of elastic energy in the periarticular bone in the interspike spaces and below the spikes and a consequent increase in the measured force of embedding. The FVU test for re-simulation was 0.02, which indicates strong correlation of the modified FE model.

## 4. Discussion

The innovative multi-spiked connecting scaffold (MSC-Scaffold) prototype gives the possibility of a new kind of biomimetic fixation of resurfacing endoprostheses in periarticular cancellous bone. The implantation method of the RHA endoprosthesis with the biomimetic MSC-Scaffold involves the spikes press-fit insertion into the trabecular bone to the defined depth, allowing the limb loading directly after the resurfacing endoprosthesis implantation. The fixation procedure of RHA endoprosthesis with the biomimetic MSC-Scaffold proceeds in two steps as follows: (1) the mechanical insertion of the endoprosthesis components into the periarticular trabecular bone on the desired osteoconductive level by the operating surgeon and (2) the adaptive bone tissue ingrowth into the interspike space of the biomimetic MSC-Scaffold.

In the present study, the state of loading of partially embedded implant (i.e., after the surgical pre-embedding) is examined. For this state, what is crucial is the determination of the critical load not causing further embedding of MSC-Scaffold during physiological loading of the operated limb. We used bone density micro-CT assessment during the mechanical embedding test of the MSC-Scaffold prototype in periarticular bone to develop a validated FE model, and therefore determined the most appropriate geometric features of the MSC-Scaffold and their values in relation to the preliminary patented version [11,12,13,14]. Similar to other works dealing with numerical modelling of bone-implant mechanical problems [19,21,22], this work combined experimental and computational analyses to perform a validation of the developed numerical model; the study was developed within the framework of two research projects (no. 4T07C05629 Polish Ministry of Science and no. NN518412638 Polish National Science Centre).

Similar to the study by Cicciù et al. [43,44], micro-CT imaging data were used to adjust and justify the 3D geometrical model and the properties of the FE model for computational simulation. FE analysis has been widely applied for simulating mechanical stress distribution in an implant and the surrounding bone [45,46,47,48]. To ensure the stability of calculations, in the built FE model, the solid element and hexahedral mesh were used; according to [43,49,50] such assumptions allowed generating a high-quality FE model.

We agree with the opinion of Marangalou et al. [51] (published in the Journal of Biomechanics) that the continuum finite element analysis has become a standard computational tool for the analysis of bone mechanical behaviour in orthopaedic biomechanics. Here, the periarticular bone was assumed to be a single-phase transversally isotropic elastic material, and the assumption regarding the material properties of the cancellous bone has been justified in [52,53,54]. According to Van Rietbergen et al. [52], since the cancellous bone trabeculae are loaded in vivo mostly in bending or compression, the longitudinal elastic modulus of cancellous bone largely determines the mechanical behaviour of this bone. However, the assumption of transversal anisotropy of cancellous bone, in general, is more adequate and it allows for analysing complex load states of this bone. Krone et al. [53] noticed, in experimental and FE analysis of the human femur, that the transversely isotropic FE model of cancellous and cortical bone material provided analysis results very similar to that obtained for the assumed orthotropic material model of both of these bone materials, in contrast to the significantly different analysis results obtained for the assumed isotropic bone model.

In the present study, the Huber–von Mises–Hencky criterion was used to determine the submaximal value of the compressive load applied to the MSC-Scaffold prototype in the experiment. In the comparative studies of Kayak et al. [55] and Tellache et al. [56], dealing with the examination of stress- and strain-based failure theories applied to femoral bone fracture prediction, it was stated that the Huber–von Mises–Hencky criterion was robust. This criterion is frequently applied for the trabecular bone failure prediction, for example in [57,58].

The FE model of the prototype MSC-Scaffold embedded in periarticular bone was validated in two experimental steps. In the first step, the mechanical load transfer at different embedding levels was investigated using the built FE model of Ti-alloy MSC-Scaffold prototype embedded in an elastic, transversely isotropic bone material. The results of numerical simulations were compared with the results of mechanical tests of embedding the MSC-Scaffold prototype in the swine periarticular bone samples. The swine experimental model has been successfully used in studies of bone mechanics [59,60] and it is a recognised animal model of choice in the case of surgical experimental implantations and mechanical testing of implants [61]. Swindle et al. [62] reported that swine and human bone have similar density and microstructure; furthermore, the biostructure of the synovial joint cartilage and the ligament system are structurally very similar in swine and human joints.

The FVU statistical test was used to determine the correlation between the obtained experimental and simulation results. In the first step of the validation procedure, we found that the proposed initial FE model did not accurately reflect the simulated phenomenon. Indeed, the obtained FVU value of 0.33 indicates the need to elucidate the reason for observed discrepancies, and to introduce changes in the initial FE model of the problem.

In the second step, the micro-CT assisted mechanical tests were performed for quantitative analysis of bone material density distribution before and during the MSC-Scaffold prototype quasi-static mechanical embedding process. Such an approach using micro-CT monitoring has also been used by other authors [37,43,49]. The micro-CT imaging data were used to modify the initial FE model by applying the simulated densified bone material insert in the FE model. It led to a strong correlation between the re-simulation and experimental results, evidenced by the FVU value reduced to 0.02. Thus, the modified FE model accurately predicts the mechanical behaviour of the investigated bone-implant system, and it can be applied for the design of material and geometric features of the considered new kind of biomimetic fixation for resurfacing endoprostheses.

The HMH stress maps demonstrated that the load transfer from the MSC-Scaffold to the bone was nearly equal, as it was expected, based on the biomimetism of the MSC-Scaffold prototype design. Thus, the MSC-Scaffold can ensure close to physiological load transfer in peri-implant bone. With the increasing distance/depth of the embedded MSC-Scaffold spikes, the share of lateral spike surface of the scaffold in the load transfer increases, resulting in lower stress values in the bone material under the spikes of the scaffold.

Our innovative MSC-Scaffold prototype provides a new kind of biomimetic entirely cementless fixation method for RA endoprostheses components. Since there are no other published FE studies, or mechanical tests, regarding surgical fixation of RA endoprostheses components, therefore, it is not possible to compare the results obtained in the present study with the results obtained by other authors.

## 5. Conclusions

1. The micro-CT assessment during the mechanical embedding test allowed for analysis of the bone material density changes directly under the MSC-Scaffold. Consequently, it enabled improvement of the initial numerical model of the considered problem by introducing a suitable simulated insert of densified bone material. This led to the significant enhancement of correlation between the re-simulation and experimental results, confirmed by the increase in the FVU test result from 0.33 to 0.02.
2. The biomimetism of the MSC-Scaffold prototype providing physiological load transfer from implant to bone was confirmed on the basis of the HMH stress maps obtained with the validated FE model of the problem.
3. In the study, the correctness of the assumptions made for the developed numerical model of the considered problem was discussed. Since a well-known and widely recognised validation method for the numerical simulation models of various problems in the field of bone-implant mechanics (being a combination of experimental studies and numerical analyses) was used, therefore:
  - the results obtained from the numerical simulation analysis of the stress distribution in periarticular bone around the MSC-Scaffold prototype partially embedded in this bone and carried out using a validated FE model can be considered to be reliable;
  - the developed validated FE model can be used in a bioengineering design of a new kind of entirely cementless biomimetic fixation of resurfacing endoprostheses, replacing degeneratively or traumatically damaged diarthrodial joints.

## Figures and Tables

**Table 1:** Mechanical properties of Ti-6Al-4V titanium alloy [26,30], the construction material of MSC-Scaffold prototypes manufactured using SLM.

Young’s Modulus (GPa)	Tensile Strength (MPa)	Yield Strength (MPa)	Elongation at Rupture (%)	Poisson’s Ratio
116	1150	1010	25	0.34

**Table 2:** The mechanical properties of the bone material used in computational studies (based on the mechanical properties of cancellous bone) [31,32,33]. *E*_1_, transverse Young’s modulus; *E*_2_, longitudinal Young’s modulus; *G*_1_, transverse shear modulus; *G*_2_, longitudinal shear modulus; *ν*_1_, transverse Poisson’s ratio; *ν_2_*, longitudinal Poisson’s ratio; *σ_c_*, ultimate compressive strength.

*E*_1_ (MPa)	*E*_2_ (MPa)	*G*_1_ (MPa)	*G*_2_ (MPa)	*ν*_1_	*ν*_2_	*σ_c_* (MPa)
608	771	260	269	0.17	0.15	25

**Table 3:** Values of marrow and soft tissue fractions during embedding in the area of interest in the bone specimen and corresponding values of trabecular bone density and longitudinal elastic modulus of bone.

Embedding Displacement (mm)	Fraction of Marrow and Soft Tissue (%)	Trabecular Bone Density (g/cm^3^)	Bone Longitudinal Elastic Modulus (MPa)
1.5	58.2	1.37	821
2	49.1	1.46	976
2.5	40.3	1.55	1173

## Data Availability

The data presented in this study are available on request from the corresponding author.
